# “Screen Time” to “AI Time”: AI Use and Cognitive, Emotional, and Behavioral Outcomes in Children and Adolescents

**DOI:** 10.2188/jea.JE20250445

**Published:** 2026-03-05

**Authors:** Abir Nagata, Kenji J. Tsuchiya

**Affiliations:** 1 Research Center for Child Mental Development, Hamamatsu University School of Medicine, Shizuoka, Japan; 2 United Graduate School of Child Development, University of Osaka, Kanazawa University, Hamamatsu University School of Medicine, Chiba University, and University of Fukui, Osaka, Japan

**Keywords:** generative AI, screen time, children and adolescents, cognitive, emotional, and behavioral outcomes

Dear Editor,

Generative artificial intelligence (AI) chatbots, including ChatGPT, Gemini, and Bing AI, have moved from novelty to ubiquity in just a few years, becoming widely used across age groups.^1^ Notably, such tools have become firmly embedded in the daily lives of many children and adolescents,^1^^,^^2^ serving purposes ranging from learning support to emotional expression and everyday conversation.^3^ Beyond offering personalized answers and a sense of being heard, these systems introduce new behavioral patterns, with both promising and concerning implications.^2^^,^^4^ Potential risks encompass a range of cognitive, emotional, and behavioral consequences, from reduced critical thinking to anxiety, depressive symptoms, and sleep disturbance.^5^ For epidemiology, this surge in AI-mediated interaction represents an emerging, measurable exposure with the capacity to influence cognitive, emotional, and social outcomes on a population scale.^6^

A growing body of evidence underscores the dual nature of this exposure. A meta-analysis of 51 studies, primarily involving students from secondary schools to university settings, reported that incorporating ChatGPT into structured educational contexts produced large improvements in learning performance and moderate gains in higher-order thinking and learning perception.^7^ In young adult populations, structured AI use has been associated with enhanced academic self-efficacy and lower anxiety, fostering engagement and emotional resilience.^8^ At the same time, excessive dependence on generative AI has been associated with diminished creativity, weaker independent problem-solving, and other adverse cognitive effects.^9^ AI chatbot dependency has also been linked to higher levels of depressive symptoms.^10^ Given early life’s heightened neuroplasticity,^11^ both the protective and harmful influences of AI use may have enduring consequences across children and adolescent developmental trajectories.^2^ The American Psychological Association^2^ stresses that AI’s impact on adolescent well-being depends on purpose, context, and individual vulnerabilities, and calls for evidence-based guidelines and monitoring frameworks to maximize benefits while reducing potential harm.

Historically, digital engagement among children and adolescents has been tracked using broad “screen time” categories, for instance, in surveys such as the Multiple Indicator Cluster Surveys^12^ and in large-scale cohort studies.^13^^,^^14^ While effective for an era dominated by passive media consumption, this framework obscures the uniquely interactive and adaptive nature of AI use. Generative AI can respond, adapt, and simulate social presence, engaging with users in ways that differ fundamentally from traditional screen-based activities.^4^ Therefore, collapsing this into generic “screen time” risks exposure misclassification and the loss of critical epidemiological insights. Without distinguishing AI use from general screen exposure, both research and policy may fail to address the specific cognitive, emotional, and behavioral impacts. However, the diversity and rapid evolution of AI systems make exposure assessment difficult. Measures such as chatbot frequency, interaction purpose (eg, academic support vs emotional expression), or reliance on AI content may soon lose relevance, underscoring the need for adaptive approaches. Despite these challenges, advancing AI-specific measurement is vital for epidemiology to capture influences of AI use in youth. We, therefore, propose three priorities:

• Incorporate AI-specific metrics into population health surveys, capturing frequency, context, and purpose of use (academic, social, emotional support) to enable nuanced dose-response analyses.
• Design and evaluate “resilience-by-design” AI tools that embed culturally relevant mental health resources, self-monitoring features, and safeguards against problematic use, tested through pragmatic trials.
• Conduct longitudinal and intervention studies to clarify causal pathways and identify moderators (eg, socioeconomic status, digital literacy) and mediators (eg, self-efficacy, peer connection) of cognitive and emotional outcomes.

Generative AI is not simply another screen-based activity; it is a rapidly spreading social and cognitive force, with profound population-level implications. There is an urgent need for epidemiology to adapt its surveillance systems, analytic frameworks, and intervention strategies so that “AI time” strengthens rather than undermines the cognitive, emotional, and social development of today’s youth.
